# To be or not to be supervisors for medical students in general practice clinical placements: a questionnaire study from Norway

**DOI:** 10.1080/02813432.2024.2337063

**Published:** 2024-04-11

**Authors:** Julie Solberg Knutsen, Gunnar Tschudi Bondevik, Steinar Hunskaar

**Affiliations:** a Department of Global Public Health and Primary Care, University of Bergen, Bergen, Norway; b National Centre for Emergency Primary Health Care, NORCE Norwegian Research Centre, Bergen, Norway

**Keywords:** Clinical clerkship, general practice, general practitioners, medical student, preceptorship, primary health care, undergraduate medical education

## Abstract

**Objective:**

Many countries experience challenges in recruiting and retaining general practitioners (GPs) as supervisors for medical students in clinical placements. We aimed to investigate the opportunities, capacities and limitations of Norwegian GPs to become supervisors.

**Design:**

Web-based cross-sectional questionnaire study.

**Setting:**

Norwegian general practice.

**Subjects:**

All GPs in Norway, including locums and those on leave, both active supervisors, and GPs who are not presently supervising medical students.

**Main outcome measures:**

GPs’ terms of salary, office facilities, limiting factors, capacity and needs for becoming or continuing as supervisors.

**Results:**

Among 5145 GPs, 1466 responded (29%), of whom 498 (34%) were active supervisors. Lack of a dedicated student office was the most reported limitation for both active supervisors (75%) and other GPs (81%). A high proportion (67%) of active supervisors reported that they could host more students per year, given financial support for equipped offices and higher salaries. With this kind of support, 48% (*n* = 461) of the GPs who were not supervisors for medical students were positive about a future supervisor role. By adjusted regression analysis, female GPs had lower likelihood of being supervisors, OR (95% CI) 0.75 (0.59–0.95) than male colleagues. GPs in the North, Mid and West regions had higher odds (OR 3.89, 3.10 and 2.42, respectively) than those in the South-East region. Teaching experience also increased the odds (2.31 (1.74–3.05).

**Conclusions:**

There seems to be capacity among both active and potential supervisors if increased salaries and financial support for office facilities are made available.

## KEY POINTS

Undergraduate training by clinical placements is important for the recruitment of doctors to general practice, and depends on a sufficient number of GPs as supervisors.

The study shows that there is sufficient capacity among Norwegian GPs to host medical students in clinical placements.

Many potential supervisors among Norwegian GPs report that they have not been approached by a university to supervise medical students.

Many supervisors state that they need increased salaries and financial support for facilities and expenses in order to supervise medical students.

## Introduction

Many countries experience challenges in recruiting and retaining general practitioners (GPs) as supervisors for medical students in clinical placements [1]. Also in Norway, medical schools find this as an increasing challenge, especially when the number of students is expanding. The shortage of GPs and a generated recruitment crisis in general practice are also central factors to these challenges [2]. There is limited knowledge about Norwegian GPs’ reported needs and opportunities to become supervisors or host more students than they do today. With this knowledge, we could better address how to maintain and recruit enough supervisors for medical students in general practice.

General practice is an important part of the medical curriculum [3], and most European universities teach theoretical general practice/family medicine [4,5]. During medical school, a broad medical knowledge and a person-centered approach to treat patients with a variety of diseases are taught through various learning situations [6]. Clinical placement in general practice is probably the most important arena where students can learn general practice and the work of GPs, and acquire the necessary understanding of the primary health care system. Workplace learning is essential to gain capability, and the quality and learning endpoints are dependent on well-trained and motivated supervisors who both challenge and support students [7].

Recruiting for a possible future career in general practice begins in medical school. Clinical placements are important showcases for students to become interested, motivated and skilled. Studies have shown associations between access to, and length of, clinical placements in general practice during medical school, and entry to postgraduate general practice training [8–11]. A study from Australia reported that students’ experience during placements had a strong correlation with the likelihood of pursuing general practice as a career later on [12]. Students consider the quality of placements as high where they can work independently, supported by good role models [13,14].

In Norway’s four medical schools, students have clinical placements in general practice of six to ten weeks duration in their 5th or 6th study year. Regulations in Norway lead medical education in a direction with increased focus on primary healthcare. There are now requirements for 10 weeks of placement in primary care, which is up to four weeks longer than the former situation [15]. The number of medical students is increasing, and there is therefore a need for more supervisors and clinical placements in general practice and primary care.

There is a limited number of scientific studies from the Nordic countries regarding clinical placements in general practice. From a Swedish student-run clinic, supervisors reported benefits in supervising related to facilities and focus on pedagogy and learning when tutoring in a student-run clinic compared with ordinary practice [16]. A study from Denmark investigated dyad practice where two students were placed together in general practice [17]. They reported that peer placements were both feasible and suitable for medical students’ first clinical stay in general practice, and that this way of structuring placements could contribute to the maintenance of the number of clinical placements. A recent Swedish study reported that intentions and capacity of individual GPs were not strong barriers to supervise medical students, and that organizational and structural barriers could be eliminated [18].

There is limited knowledge on Norwegian GPs’ prerequisites and needs to become or continue as supervisors. This is important in terms of retaining and developing the current clinical placement system. This study aimed to investigate Norwegian GPs’ willingness, capacity and limitations for supervising medical students in clinical placements. Our hypothesis was that there is both willingness and positive attitudes among the GPs to host medical students in clinical placements, but that there are economical and practical obstacles. We explored both current supervisors and GPs who are not presently supervising medical students.

## Materials and methods

We developed a web-based questionnaire which was sent to all regular GPs in Norway in February 2022. The survey was made using the Questback Essentials software. The questionnaire was accessible for one month, and three reminders were sent during that period.

### Subjects

In collaboration with the Norwegian College of General Practice, the questionnaire was sent by email to all their members, which among others included the target group; all regular GPs in Norway (*n* = 5145 per January 2022). Other members could be retired or former GPs, or doctors from other specialties working in primary care. These were excluded after confirming the respondents’ occupational status at the beginning of the questionnaire.

### The questionnaire

The email contained a link to the questionnaire and had an introductory text stating that the target group was all regular GPs, including locum GPs and GPs on intermediate leave.

The questionnaire consisted of 40 questions divided into three sections. This article presents results from the first and second section. The first section included anonymous background data about the respondents. The second section mapped willingness, capacity and limitations of being a supervisor.

The demographic variables consisted of gender (male, female, other), age group (<36, 36–45, 46–55, >55 years), specialization status (approved specialist in general practice, ongoing graduate training in general practice, other), geographical region (North, Middle, West or South-East regions of Norway), corresponding to the areas covered by the four universities with a medical school. Size of the municipality where the respondent worked was categorized according to the number of inhabitants, <5000, 5001–20,000, 20,001–50,000, 50,001–100,000 and >100,000. These categories were determined to ensure a sufficient number of people in each group to prevent identification of respondents. The questionnaire also included variables about the number of GPs working in the surgery (numeric scale), supervisor status for interns/residents and experience in research and teaching (yes/no). For the logistic regression analysis, teaching experience and supervisor experience for interns/residents were merged into one variable called ‘Teaching experience’.

The second section of the questionnaire initiated with asking whether the GP was a supervisor for medical students or not: ‘Have you during the last three years (after January 1st, 2019) been supervising 5th/6th year medical students in clinical placements?’. The answer (yes/no) divided the respondents into two groups (active supervisors and non-supervisors)with many similar and some slightly different questions.

If the answer was ‘yes’, a question regarding the students’ university of origin with five alternatives followed (four Norwegian universities or studying abroad). GPs were asked if they had received a fixed salary from the university or whether they were paid per student. Limitations for hosting students, such as lack of office and equipment for the student, were mapped with four alternatives and a free text option. The GPs’ teaching capacity was investigated by their ability to host more students simultaneously or several times per year (yes/no) and what was eventually needed to do so (alternatives and free text option).

For the respondents who did not have experience as supervisors, the questions were adapted to this situation. In addition, they were asked if they had ever received an invitation from a university to become a supervisor (yes/no).

The questionnaire was piloted twice. First, as an internal pilot with academic colleagues with different professional backgrounds, including GPs and clinical placement supervisors. The second pilot was an external completion of the questionnaire with employees at a GP surgery. Based on the pilots, one question was added, and some questions were reformulated. The questionnaire was estimated to take five to ten minutes to complete. To compare our responder population with the total population of GPs, we used demographic data obtained from The Norwegian Medical Association.

### Statistical analysis

The data were analyzed using descriptive statistics with frequencies (%). Multivariate logistic regression was performed to assess the impact of the included variables on the likelihood of being a supervisor for medical students in clinical placements, using a 95% confidence interval. Statistical analysis was performed using IBM SPSS Statistics, version 27 (Armonk, NY).

### Ethical considerations

An application for approval was sent to the Regional Committee for Medical and Health Research Ethics of Western Norway. The committee assessed the project as an exception for formal ethical approval (ref. 434056). The respondents were anonymous, and the data were registered in the software account without any links to the respondents.

## Results

**Figure 1.**
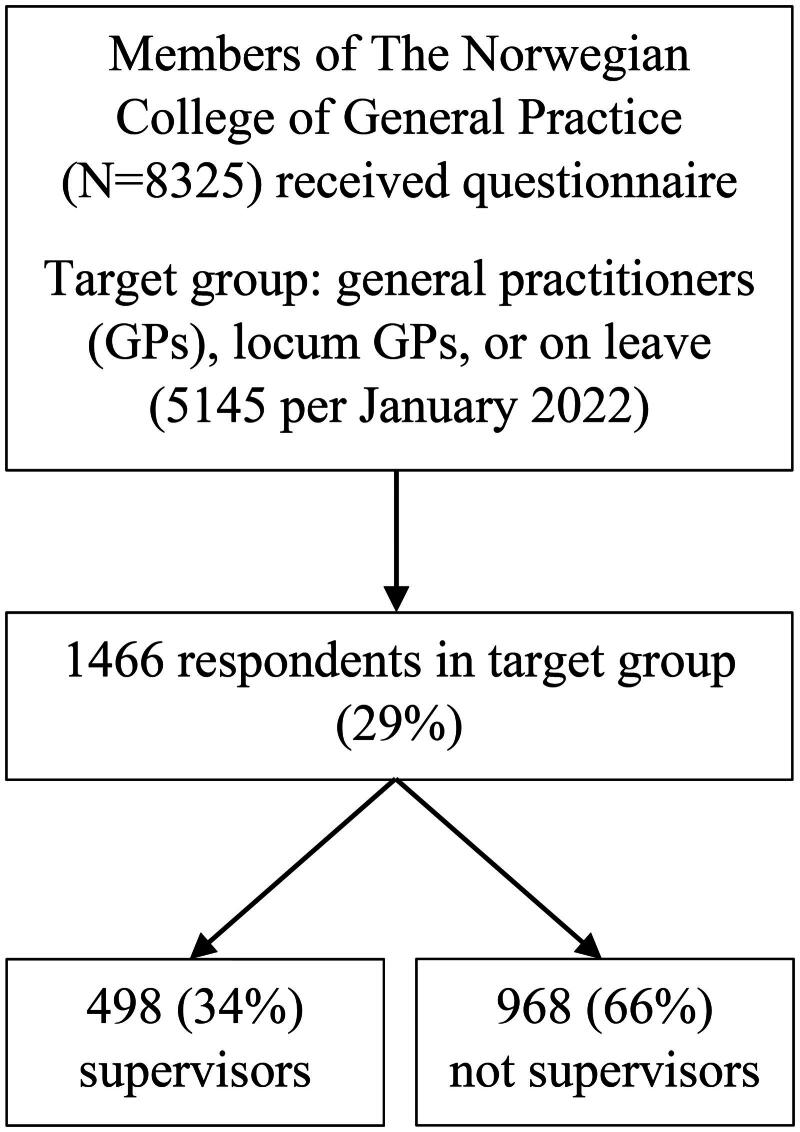
Flowchart of data collection.

We received 1466 responses from members of the target group, representing 29% of the Norwegian regular GPs in January 2022. Among the respondents, 498 (34%) stated that they had been supervisors for medical students during the last three years, while 968 (66%) had not (Figure 1).

Table 1 presents characteristics of the study participants. Except for the age group under 36 years, there was an even distribution and proportion of supervisors in the different age groups. Half of the respondents worked in the South-East region, and 59% of the GPs who were not supervisors for medical students, were also from this region. The smallest municipalities (<5000 inhabitants) had the lowest response rate, but the highest proportion of supervisors (59/143 = 41%). The majority were specialists in general practice and worked in a surgery with four to six GPs. More than half of the respondents had experience in supervising interns or residents in general practice.

**Table 1.** Characteristics of the study participants by supervisor status.

	All respondents		Supervisors^a^			
	*N* = 1466		Yes *N* = 498 (34%)		No *N* = 968 (66%)	
	*n*	%	*n*	%	*n*	%
Gender						
Male	686	48	261	54	425	45
Female	734	52	219	46	515	55
Age group (years)						
<36	224	16	54	11	170	18
36–45	503	35	182	37	321	33
46–55	344	24	123	25	221	23
>55	388	27	136	28	252	26
Region						
North	185	13	93	19	92	10
Mid	212	15	104	21	108	11
West	330	23	134	27	196	20
South-East	734	50	164	33	570	59
Municipality size (inhabitants)						
<5000	143	10	59	12	84	9
5001–20,000	352	24	112	23	240	25
20,001–50,000	338	23	103	21	235	24
50,001–100,000	209	14	71	14	138	14
>100,000	420	29	152	31	268	28
Specialist in general practice						
Yes	1040	71	395	80	645	67
Under specialization	380	26	97	20	283	29
Other	43	3	5	1	38	4
Size of surgery (doctors)						
1–3	370	25	114	23	256	27
4–6	808	55	280	56	528	55
7+	284	19	103	21	181	19
Interns in practice						
Yes	770	53	289	58	481	50
No	691	47	207	42	484	50
Teaching experience						
Yes	452	31	242	49	210	22
No	1003	69	253	51	750	78
Research experience						
Yes	226	16	94	19	132	14
No	1214	84	392	79	822	85
Research without authorship						
Yes	203	14	90	19	113	12
No	1238	86	397	82	841	88
Ph.D. and ongoing research						
Yes	65	5	29	6	36	4
No	1375	96	456	94	919	96
Supervisor for interns or residents						
Yes	773	53	320	65	453	47
No	688	47	176	36	512	53

Missing 3–46 (0–3%) in the categories.

^a^ Supervisor Yes were the GPs who reported being a supervisor for 5th or 6th-years medical students at the GP office in the period 2019–2021.

Table 2 presents the respondents’ preferences for salary terms, office facilities and limitation factors for being supervisors. A majority (61%) of active supervisors were paid per student (fee for service) while 57% of the respondents who were not supervisors preferred fixed salary. Among active supervisors, 40% had a dedicatedly equipped student office, and 12% had no suitable consultation room for students at all. Among the GPs who were not supervisors, almost one-third had no suitable room for the student and half of the GPs could offer alternating consultation rooms. We found that the proportion of GPs having a ‘Designated student office the whole period’ was three times higher in the North compared to the West region. For limiting factors, the answers from supervisors/non-supervisors were rather similar. Out of four alternatives listed, limited number of offices was the most common alternative (75% of supervisors and 81% of non-supervisors).

**Table 2.** Salary terms, facilities and limitations by supervisor status.

	Supervisors^a^			
	Yes *N* = 498 (34%)		No *N* = 968 (66%)	
Salary agreement/wanted salary terms	*n*	%	*n*	%
Fixed salary	181	39	546	57
Paid per student	284	61	413	43
Facilities and room for student				
Designated student office the whole period	199	40	138	14
Students (would) alternate between vacant offices	224	45	495	51
(Would) use other room^b^	15	3	71	7
Have no suitable room for student	59	12	264	27
Limiting factors				
Limited number of offices	285	75	718	81
Space limitations	93	25	214	24
Lack of computer/software	40	11	137	16
Lack of equipment	34	9	109	12

^a^ Supervisors Yes were the GPs who reported being supervisor*s* for 5th or 6th-years medical students at the GP office in the period 2019–2021, and they answered based on their current situation. The GPs not supervising medical students in that period (no), answered based on desires if they were to be supervisors for medical students.

^b^ Other room could be a not fully equipped room like ECG-room or room for Chirurgia minor.

Factors regarding active and potential supervisors’ capacity on hosting students are presented in Table 3. Some supervisors (14%) hosted more than one student at the same time. One in five stated that this could be a possibility in the future. Improved facilities like student office and increased salaries, were the highest ranked needs to do so. For the willingness of hosting more students per year, two-thirds of the active supervisors stated that this might be possible. Higher salaries, more offices, and support for equipment and costs were reported as main needs.

**Table 3.** Capacity and needs reported by GPs according to supervisor status.

Active supervisors^a^ *N* = 498 (34%)		
	*n*	%
Currently hosting more than one student at the same time	70	14
Could in the future host more students simultaneously	99	20
Needs to host several students simultaneously		
More offices	342	70
Higher salaries	291	60
Economically support for equipment and office	216	44
Increased competence in clinical teaching	91	19
More equipment	86	18
Closer follow-up by the university	55	11
Do not know	16	3
Could in the future host further more students during a year	330	67
Needs to host further more students during a year		
Higher salaries	292	62
More offices	232	49
Economically support for equipment and office	181	38
Increased competence in clinical teaching	105	22
Closer follow-up by the university	67	14
More equipment	55	12
Do not know	34	7
Not supervisors *N* = 968 (66 %)		
	*n*	%
Received invitation to become supervisor?		
Yes	247	29
No	683	71
Become a supervisor in the future?		
Yes	461	48
No	166	17
Maybe	334	35
Needs for hosting medical student in the future		
More offices	590	61
Economically support for equipment and office	476	50
Increased competence in clinical teaching	298	31
Close follow-up by the university	197	21
More equipment	158	16
Do not know	50	5

^a^ Active supervisors if supervising 5th or 6th-years medical students in Norway between 2019 and 2021.

Of the GPs who were not supervisors, a majority (*n* = 683, 71%) reported that they had not received any invitation to become a supervisor, while nearly a half of them (*n* = 461) were positive to become so in the future. More offices and economic support for equipment and facilities were the highest ranked needs to take on the task, but one-third also stated a need for increased competence in clinical teaching.

Table 4 presents the logistic regression for being a supervisor for medical student in clinical placement by demographics and participant characteristics. Four of the predictors made a statistically significant contribution to the model (gender, region, specialist status and teaching experience). The strongest predictor for being a supervisor for medical students was working in the North, Mid or West regions compared with the South-East region, with the following odds ratios (95% CI) 3.89 (2.63–5.75), 3.10 (2.18–4.38) and 2.42 (1.79–3.28), respectively. This indicated that respondents working in these regions were two to four times more likely to be supervisors for medical students than those living in the South-East region, adjusted for other variables. Having former teaching and supervisor experience made it over two times more likely to be a supervisor for medical students, with OR 2.31 (1.74–3.05). This did not include supervising medical students in general practice clinical placements, but for example supervising interns or residents. Female GPs had lower odds of being a supervisor for medical students with OR 0.75 (0.59–0.95). Being specialist in general practice made it more likely to be a supervisor compared to those under specialization, OR 0.72, (0.50–1.04) and those with other specialties, OR 0.15, (0.04–0.51).

**Table 4.** Likelihood of being a supervisor for medical student in clinical placement. Logistic regression with crude and adjusted results. Missing: 71

	Respondents	Crude		Adjusted	
	*n*	OR	(95% CI)	OR	(95% CI)
Gender					
Male	686	Ref.		Ref.	
Female	734	0.69	(0.56–0.86)*	0.75	(0.59–0.95)*
Age group (years)					
<36	224	Ref.		Ref.	
36–45	503	1.79	(1.25–2.55)*	1.26	(0.82–1.95)
46–55	344	1.75	(1.20–2.56)*	1.19	(0.73–1.93)
>55	388	1.70	(1.17–2.46)*	1.15	(0.70–1.89)
Region					
South-East	734	Ref.		Ref.	
North	185	3.51	(2.51–4.92)**	3.89	(2.63–5.75)**
Mid	212	3.35	(2.43–4.61)**	3.10	(2.18–4.38)**
West	330	2.38	(1.80–3.14)**	2.42	(1.79–3.28)**
Municipality size (inhabitants)					
<5000	143	Ref.		Ref.	
5001–20,000	352	0.66	(0.45–0.99)*	0.80	(0.50–1.28)
20,001–50,000	338	0.62	(0.42–0.94)*	0.90	(0.56–1.46)
50,001–100,000	209	0.73	(0.47–1.14)	1.05	(0.64–1.74)
>100,000	420	0.81	(0.55–1.19)	1.13	(0.70–1.81)
Specialist in general practice					
Yes	1040	Ref.		Ref.	
Under specialization	380	0.56	(0.43–0.73)**	0.72	(0.50–1.04)
Other	43	0.22	(0.08–0.55)*	0.15	(0.04–0.51)*
Size of surgery (doctors)					
1–3	370	0.84	(0.65–1.09)	1.05	(0.78–1.42)
4–6	808	Ref.		Ref.	
7+	284	1.07	(0.81–1.42)	1.00	(0.73–1.38)
Teaching experience^a^					
No	510	Ref.	Ref.	Ref.	
Yes	942	2.80	(2.18–3.61)**	2.31	(1.74–3.05)**

* *p* < .05.

** *p* < .001.

^a^ Teaching experience and supervisor experience are merged into ‘Teaching experience’ and does not include the experience of supervising medical students in clinical placement in general practice.

## Discussion

The respondents reported a large potential for more supervisors among GPs in Norway, both by recruiting new ones and expanding the number of students hosted by the existing supervisors. There is, however, a strong message from the GPs that increased salaries and financial support for more student offices are necessary. Among the GPs not being supervisors, a large majority reported not to have received invitation to become so. The regression analysis showed that male GPs, specialists, and those with teaching and supervising experience had greater odds of being supervisors for medical students during placements. GPs in the South-East region had lower odds of being supervisors compared with the other regions.

### Strengths and limitations

There was a relatively low response rate of 29%. However, the number of respondents in each group was large enough both for statistical power and for estimating the potential national supervisor capacity among GPs. Although there is a potential selection bias among the respondents, our data indicate satisfactory capacity for supervisors in the future.

We developed our own questionnaire which may have affected the validity of the study, as there could be factors that we did not consider when making alternatives. Although the authors come from an experienced GP research group, the validity could have been increased by a more formal development phase. Modifications based on feedback from both internal and external pilots, strengthened the questionnaire.

We sent the questionnaire to all GPs in Norway individually instead of contacting each GP surgery to have a combined answer per surgery. We know that many GPs share the responsibility for the student during a placement, and a student may thereby get supervision from more GPs than the one titled student supervisor in the university registry. Therefore, we probably have answers from GPs who have experience as supervisors but have not yet signed a contract with the university. This could have affected the data, especially on questions about office facilities. If, for instance, three GPs from the same surgery answered that the students have their own office, and they all mean the same office, there could be an overestimation of available student offices. To ensure anonymity, we did not ask about surgery affiliation, which prevented us from sorting out this bias. There was considerable regional variation in the results of this specific question.

Comparing our study population with the total Norwegian population of GPs, we see similar distributions on demographics. However, we found a somewhat higher proportion of specialists answering our questionnaire than in the total population of GPs. In future studies, it will be of value to include variables that can identify representativity factors.

### Findings in relation to other studies

A comparable American study found that monetary payment (honorarium or as compensation for loss of income) was important for half of the respondents [19]. In our study, 60% of the active supervisors stated higher salaries as a need for hosting more students simultaneously or per year. The current payment in Norway is approximately EUR 100 per day per student, with some variance between medical schools. Most GPs in Norway are self-employed within a fee for service system, and compensation for loss of income is important. An hour of patient treatment usually pays better off economically than supervising students. One could consider whether student supervision should be as profitable as clinical work. Nevertheless, our results reflect that monetary payment could play an important role in both retaining and recruiting more supervisors in general practice.

The need for and lack of offices designated for the students was an important finding in our study, as it was in a study from England, which found time and space limitations as top ranking obstacles for teaching undergraduates in a general practice setting [20]. Teaching rooms are reported as a limitation in clinical education for medical students placed both in hospital and general practice settings [21]. For the best learning results, the students need room where they can perform a properly consultation with the patient. In Norwegian general practice, differences in size of group practices could affect the space limitation. In our study population, half of the respondents worked in a group practice with four to six GPs, and only 19% worked in a group practice with seven or more GPs. In larger health care centers, space limitations may not be a major problem, while the infrastructure of a GP surgery with four to six GPs could be a barrier for available rooms. However, this was not supported by our findings.

A recent study in Swedish primary health care found that the intentions and capacity of individual supervisors were not strong barriers to supervise medical students [18]. This correlates with our findings on capacity, as many of the GPs in Norway also could either host more students or become supervisors in the future.

### Implications and meaning of the study

In a time when recruitment to general practice is critical, and the shortage of GPs is about to threaten the primary health care system in Norway, different ways of recruiting medical graduates to general practice are needed. The association between undergraduate clinical placements and choosing general practice as a career is scientifically strong. The evidence from the present study regarding GPs’ capacity, willingness and limitations for hosting students indicates specific challenges to be solved for retaining and recruiting GPs to be supervisors during undergraduate training. GPs need economic support to provide equipped offices for the students, and they need higher salaries for the job as supervisors to compensate for loss of income. These incentives are upstream approaches contributing to more GPs in Norway in the future.

The results of the present study provide a good overview of the most common limitations and needs to host more medical students in general practice in Norway. There are individual needs for support within a surgery, and it is therefore necessary to have tailored agreements with each GP who wants to supervise medical students. Further studies are needed to investigate how to increase the number of clinical placements. The use of peer-placement (i.e. two students per supervisor, simultaneously) in general practice could also be further explored to cope with the capacity challenge medical schools are facing.
